# 6,6′-Di-*tert*-butyl-4,4′-dimeth­oxy-2,2′-[1,3-diazinane-1,3-diylbis(methyl­ene)]diphenol 0.19-hydrate

**DOI:** 10.1107/S1600536811053542

**Published:** 2011-12-21

**Authors:** Augusto Rivera, Derly Marcela González, Jaime Ríos-Motta, Karla Fejfarová, Michal Dušek

**Affiliations:** a Departamento de Química, Universidad Nacional de Colombia, Ciudad Universitaria, Bogotá, Colombia; b Institute of Physics ASCR, v.v.i., Na Slovance 2, 182 21 Praha 8, Czech Republic

## Abstract

In the title hexa­hydro­pyrimidine derivative, C_28_H_42_N_2_O_4_·0.19H_2_O, the 1,3-diazinane ring has a chair conformation with a diequatorial substitution. The asymmetric unit contains one half-organic mol­ecule and a solvent water mol­ecule with occupany 0.095. The mol­ecule lies on a mirror plane perpendicular to [010] which passes through the C atoms at the 2- and 5-positions of the heterocyclic system. The partially occupied water mol­ecule is also located on this mirror plane. The dihedral angle between the planes of the aromatic rings is 17.71 (3)°. Two intra­molecular O—H⋯N hydrogen bonds with graph-set motif *S*(6) are present. No remarkable inter­molecular contacts exist in the crystal structure.

## Related literature

For a related structure, see: Rivera *et al.* (2012*a* ▶). For the synthesis of the precursor, see: Rivera *et al.* (2010 ▶). For the preparation of the title compound, see: Rivera *et al.* (2012*b* ▶). For bond-length data, see: Allen *et al.* (1987 ▶). For puckering parameters, see: Cremer & Pople (1975 ▶). For hydrogen-bond graph-set nomenclature, see: Bernstein *et al.* (1995 ▶).
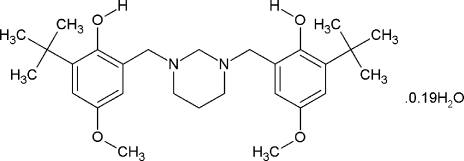


## Experimental

### 

#### Crystal data

C_28_H_42_N_2_O_4_·0.19H_2_O
*M* *_r_* = 473.5
Orthorhombic, 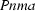

*a* = 8.2265 (1) Å
*b* = 33.0103 (2) Å
*c* = 10.0322 (5) Å
*V* = 2724.34 (14) Å^3^
*Z* = 4
Cu *K*α radiation
μ = 0.61 mm^−1^
*T* = 120 K
0.42 × 0.36 × 0.30 mm

#### Data collection

Agilent Xcalibur diffractometer with an Atlas (Gemini ultra Cu) detector
Absorption correction: multi-scan (*CrysAlis PRO*; Agilent, 2010 ▶) *T*_min_ = 0.073, *T*_max_ = 1
54017 measured reflections
2456 independent reflections
2353 reflections with *I* > 3σ(*I*)
*R*_int_ = 0.027

#### Refinement

*R*[*F* ^2^ > 2σ(*F* ^2^)] = 0.035
*wR*(*F* ^2^) = 0.122
*S* = 2.64
2456 reflections
164 parameters
H atoms treated by a mixture of independent and constrained refinement
Δρ_max_ = 0.17 e Å^−3^
Δρ_min_ = −0.14 e Å^−3^

### 

Data collection: *CrysAlis PRO* (Agilent, 2010 ▶); cell refinement: *CrysAlis PRO*; data reduction: *CrysAlis PRO*; program(s) used to solve structure: *SIR2002* (Burla *et al.*, 2003 ▶); program(s) used to refine structure: *JANA2006* (Petříček *et al.*, 2006 ▶); molecular graphics: *DIAMOND* (Brandenburg & Putz, 2005 ▶); software used to prepare material for publication: *JANA2006*.

## Supplementary Material

Crystal structure: contains datablock(s) global, I. DOI: 10.1107/S1600536811053542/go2039sup1.cif

Structure factors: contains datablock(s) I. DOI: 10.1107/S1600536811053542/go2039Isup2.hkl

Additional supplementary materials:  crystallographic information; 3D view; checkCIF report

## Figures and Tables

**Table 1:** Hydrogen-bond geometry (Å, °)

*D*—H⋯*A*	*D*—H	H⋯*A*	*D*⋯*A*	*D*—H⋯*A*
O1—H1⋯N1	0.890 (15)	1.843 (15)	2.6735 (10)	154.6 (14)

**Fig. 1.**
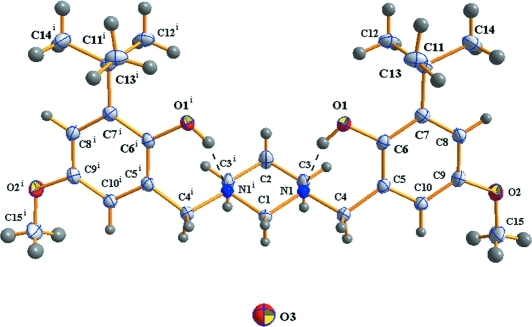
A view of (I) with the numbering scheme. Displacement ellipsoids are drawn at the 50% probability level.

**Fig. 2.**
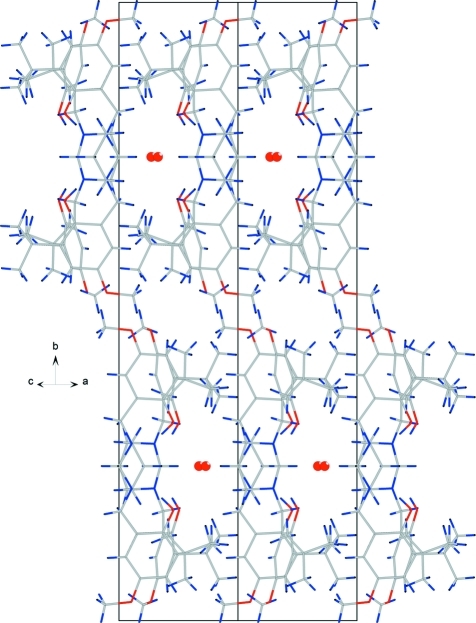
The packing of (I), viewed along the [1,0,1] axis.

## supplementary crystallographic information

### Comment

The asymmetric unit (Fig. 1), contains a one symmetry independent half molecule of 2,2'-(dihydropyrimidine-1,3(2*H*,4*H*)-diyldimethanediyl)bis(6-tertbutyl-4-methoxyphenol) and a solvent water molecule with occupany 0.095. The molecule lies on a mirror plane perpendicular to [0,1,0] which passes through the central C atom of the heterocyclic system. In Fig. 1 primed atoms were positioned on the other half of the molecules and had symmetry codes (*x*, 1/2 - *y*, *z*). The hexahydropyrimidine ring of the title compound adopts a chair conformation with a diequatorial substitution (Cremer & Pople, 1975) with puckering parameters Q, θ and φ of 0.5891 (10) Å, 3.01 (11)°, 60.0 (18)°. In the molecule of the title compound (Fig. 1), bond lengths (Allen *et al.*, 1987) and angles are normal and comparable to the related structure namely 2,2'-(dihydropyrimidine-1,3(2*H*,4*H*)-diyldimethanediyl)bis (6-methylphenol) whose crystallographic data have been deposited at the Cambridge Crystallographic Data Center. The CCDC deposition number is 854735 (Rivera *et al.*,2012*a*). However a careful comparison with the values of the corresponding angles and bond distances in the related structure (Rivera,*et al.* 2012*a*), indicated that the O1—C6—C7 angle increase by 1.82°. The crystal structure of the title confirms the presence of two O—H···N(1,3-diazinane) hydrogen bond with graph-set motif S(6) (Bernstein *et al.* 1995) (Table 1). The N···O distance [N1···O1, 2.6735 (10) Å] is shorter in comparison with the values observed in related structure (Rivera, *et al.* 2012*a*), showing a slightly increase in hydrogen-bonding strength.

The most obvious difference between the title compound and the related structure (Rivera, *et al.* 2012*a*) is the presence of mirror symmetry in the solid state with molecules bisected by mirror planes (the C1 and C2 atoms of the 1,3-diazinane ring lie on the mirror plane). The partially occupied water molecule also is located on this mirror plane. Another important difference is observed in the dihedral angle between the phenyl rings, which is -17.711 (30)° for the title compound and 58.431 (38)° for related structure (Rivera, *et al.* 2012*a*). The deviation of the dihedral angle in (I) is probably due to repulsive interactions between the *tert*-butyl groups.

Fig 2. shows the crystal packing with channels extended along the [1,0,1] axis and accommodating the water molecules. Each channel is composed of two symmetry equivalent positions of the organic molecule. No remarkable intermolecular contacts exist in the presented structure.

### Experimental

The title compound was obtained according to our recently reported methodology (Rivera *et al.*, 2012*b*), that is, to a stirred solution of 2-*tert*-butyl-4-methoxy-phenol (2.0 mmol) in 96% ethanol (5 ml) heated under reflux, was added slowly a solution of 1,3,7,9,13,15,19,21-octaazapentacyclo[19.3.1.^13,7^.1^9,13^.1^15,19^]octacosane prepared according to a previous report (Rivera *et al.*, 2010) (200 mg, 0.54 mmol) in 96% ethanol (5 ml). Upon completion of the addition, the reaction mixture was stirred under reflux for 60 h. Then the reflux was stopped, the solvent was removed on a rotary evaporator under vacuum and the residue obtained was chromatographed on silica gel eluting with benzene/AcOEt (gradient elution with 5% to 20% AcOEt) to afford a solid which was recrystallized in 96% ethanol to provide high quality crystals of the title compound (I), (Yield 27.0%, m.p. 403–404 K).

### Refinement

All hydrogen atoms were discernible in difference Fourier maps and could be refined to reasonable geometry. According to common practice the hydrogen atoms attached to carbons were kept in ideal positions with C–H distance 0.96 Å during the refinement. The methyl H atoms were allowed to rotate freely about the adjacent C—C bonds. The coordinates of the hydrogen atom bonded to oxygen were refined freely. All H atoms were refined with displacement coefficients *U*_iso_(H) set to 1.5*U*_eq_(C, O) for the methyl- and hydroxyl groups and to 1.2Ueq(C) for the CH–, and CH_2_– groups.

### Crystal data

**Table:**

C_28_H_42_N_2_O_4_·0.19H_2_O	*F*(000) = 1029.6
*M**_r_* = 473.5	*D*_x_ = 1.154 Mg m^−^^3^
Orthorhombic, *P**n**m**a*	Cu *K*α radiation, λ = 1.5418 Å
Hall symbol: -P 2ac 2n	Cell parameters from 37659 reflections
*a* = 8.2265 (1) Å	θ = 4.0–67.0°
*b* = 33.0103 (2) Å	µ = 0.61 mm^−^^1^
*c* = 10.0322 (5) Å	*T* = 120 K
*V* = 2724.34 (14) Å^3^	Block, colourless
*Z* = 4	0.42 × 0.36 × 0.30 mm

### Data collection

**Table:**

Agilent Xcalibur diffractometer with an Atlas (Gemini ultra Cu) detector	2456 independent reflections
Radiation source: Enhance Ultra (Cu) X-ray Source	2353 reflections with *I* > 3σ(*I*)
mirror	*R*_int_ = 0.027
Detector resolution: 10.3784 pixels mm^-1^	θ_max_ = 67.1°, θ_min_ = 4.6°
Rotation method data acquisition using ω scans	*h* = −9→9
Absorption correction: multi-scan (*CrysAlis PRO*; Agilent, 2010)	*k* = −39→39
*T*_min_ = 0.073, *T*_max_ = 1	*l* = −11→11
54017 measured reflections	

### Refinement

**Table:**

Refinement on *F*^2^	85 constraints
*R*[*F*^2^ > 2σ(*F*^2^)] = 0.035	H atoms treated by a mixture of independent and constrained refinement
*wR*(*F*^2^) = 0.122	Weighting scheme based on measured s.u.'s *w* = 1/(σ^2^(*I*) + 0.0016*I*^2^)
*S* = 2.64	(Δ/σ)_max_ = 0.005
2456 reflections	Δρ_max_ = 0.17 e Å^−^^3^
164 parameters	Δρ_min_ = −0.14 e Å^−^^3^
0 restraints	

### Special details

**Table:**

Experimental. CrysAlisPro, Agilent (2010), Empirical absorption correction using spherical harmonics, implemented in SCALE3 ABSPACK scaling algorithm.
Refinement. The refinement was carried out against all reflections. The conventional *R*-factor is always based on *F*. The goodness of fit as well as the weighted *R*-factor are based on *F* and *F*^2^ for refinement carried out on *F* and *F*^2^, respectively. The threshold expression is used only for calculating *R*-factors *etc*. and it is not relevant to the choice of reflections for refinement.The program used for refinement, Jana2006, uses the weighting scheme based on the experimental expectations, see _refine_ls_weighting_details, that does not force *S* to be one. Therefore the values of *S* are usually larger than the ones from the *SHELX* program.

### Fractional atomic coordinates and isotropic or equivalent isotropic displacement parameters (Å^2^)

**Table:**

	*x*	*y*	*z*	*U*_iso_*/*U*_eq_	Occ. (<1)
O1	0.48228 (8)	0.17951 (2)	0.02311 (7)	0.0238 (2)	
O2	0.18527 (9)	0.03041 (2)	0.08155 (9)	0.0348 (3)	
O3	0.2004 (8)	0.25	0.5283 (7)	0.040 (3)*	0.185 (6)
N1	0.20147 (9)	0.21389 (2)	0.09520 (8)	0.0196 (3)	
C1	0.17203 (15)	0.25	0.17490 (13)	0.0188 (4)	
C2	0.1074 (2)	0.25	−0.10270 (15)	0.0302 (4)	
C3	0.08755 (13)	0.21221 (2)	−0.01750 (10)	0.0253 (3)	
C4	0.19236 (11)	0.17724 (3)	0.17868 (10)	0.0219 (3)	
C5	0.26169 (12)	0.14026 (3)	0.11072 (10)	0.0196 (3)	
C6	0.40731 (11)	0.14256 (3)	0.03904 (9)	0.0189 (3)	
C7	0.47830 (11)	0.10754 (3)	−0.01656 (9)	0.0197 (3)	
C8	0.39696 (12)	0.07106 (3)	0.00193 (10)	0.0226 (3)	
C9	0.25098 (12)	0.06858 (3)	0.07237 (10)	0.0235 (3)	
C10	0.18330 (11)	0.10307 (3)	0.12733 (10)	0.0211 (3)	
C11	0.64179 (11)	0.10927 (3)	−0.09068 (10)	0.0214 (3)	
C12	0.63025 (13)	0.13705 (3)	−0.21310 (10)	0.0273 (3)	
C13	0.77305 (12)	0.12524 (3)	0.00455 (10)	0.0271 (3)	
C14	0.69608 (13)	0.06750 (3)	−0.13975 (12)	0.0323 (3)	
C15	0.04374 (13)	0.02586 (3)	0.16101 (14)	0.0390 (4)	
H1	0.4062 (18)	0.1977 (5)	0.0432 (14)	0.0357*	
H1a	0.243304	0.25	0.250666	0.0225*	
H1b	0.061463	0.25	0.205494	0.0225*	
H2a	0.213383	0.25	−0.142824	0.0362*	
H2b	0.027077	0.25	−0.17208	0.0362*	
H3a	−0.021813	0.210856	0.015603	0.0304*	
H3b	0.11002	0.188634	−0.070414	0.0304*	
H4a	0.081224	0.172204	0.202722	0.0263*	
H4b	0.248999	0.181858	0.260948	0.0263*	
H8	0.442701	0.046769	−0.034967	0.0271*	
H10	0.083356	0.101481	0.176454	0.0253*	
H12a	0.59744	0.16367	−0.185502	0.041*	
H12b	0.734431	0.138538	−0.255938	0.041*	
H12c	0.551668	0.126314	−0.274392	0.041*	
H13a	0.751922	0.153186	0.024643	0.0407*	
H13b	0.771525	0.109711	0.085441	0.0407*	
H13c	0.877797	0.122826	−0.036879	0.0407*	
H14a	0.796577	0.070049	−0.187653	0.0484*	
H14b	0.711271	0.04983	−0.064775	0.0484*	
H14c	0.614427	0.056424	−0.197596	0.0484*	
H15a	0.010043	−0.001985	0.160156	0.0585*	
H15b	0.067158	0.033969	0.25087	0.0585*	
H15c	−0.041734	0.042506	0.125745	0.0585*	

### Atomic displacement parameters (Å^2^)

**Table:**

	*U*^11^	*U*^22^	*U*^33^	*U*^12^	*U*^13^	*U*^23^
O1	0.0244 (4)	0.0180 (4)	0.0289 (5)	−0.0026 (3)	0.0051 (3)	−0.0005 (3)
O2	0.0366 (5)	0.0176 (4)	0.0502 (6)	−0.0053 (3)	0.0152 (3)	−0.0021 (3)
N1	0.0249 (4)	0.0159 (4)	0.0180 (5)	0.0012 (3)	0.0013 (3)	0.0002 (3)
C1	0.0219 (6)	0.0169 (6)	0.0175 (7)	0	0.0009 (5)	0
C2	0.0451 (9)	0.0234 (7)	0.0220 (8)	0	−0.0092 (6)	0
C3	0.0336 (6)	0.0185 (5)	0.0239 (5)	−0.0007 (4)	−0.0056 (4)	−0.0031 (4)
C4	0.0271 (5)	0.0174 (5)	0.0213 (5)	0.0011 (3)	0.0042 (4)	0.0025 (4)
C5	0.0220 (5)	0.0188 (5)	0.0180 (5)	0.0020 (3)	−0.0016 (3)	0.0024 (3)
C6	0.0207 (5)	0.0183 (5)	0.0176 (5)	−0.0007 (3)	−0.0023 (4)	0.0018 (3)
C7	0.0203 (5)	0.0220 (5)	0.0167 (5)	0.0022 (3)	−0.0025 (3)	0.0004 (3)
C8	0.0254 (5)	0.0188 (5)	0.0236 (5)	0.0035 (4)	0.0001 (4)	−0.0011 (4)
C9	0.0263 (5)	0.0181 (5)	0.0263 (6)	−0.0020 (4)	−0.0001 (4)	0.0022 (4)
C10	0.0205 (5)	0.0208 (5)	0.0221 (5)	0.0007 (3)	0.0008 (4)	0.0034 (3)
C11	0.0210 (5)	0.0239 (5)	0.0195 (5)	0.0012 (4)	0.0011 (4)	−0.0010 (4)
C12	0.0244 (5)	0.0374 (5)	0.0202 (5)	0.0025 (4)	0.0024 (4)	0.0034 (4)
C13	0.0199 (5)	0.0395 (6)	0.0220 (5)	0.0003 (4)	0.0005 (4)	−0.0012 (4)
C14	0.0290 (5)	0.0293 (5)	0.0386 (7)	0.0041 (4)	0.0097 (5)	−0.0059 (4)
C15	0.0367 (6)	0.0233 (5)	0.0570 (8)	−0.0070 (4)	0.0153 (5)	0.0034 (5)

### Geometric parameters (Å, °)

**Table:**

O1—C6	1.3762 (11)	C7—C8	1.3899 (13)
O1—H1	0.890 (15)	C7—C11	1.5379 (13)
O2—C9	1.3743 (12)	C8—C9	1.3958 (14)
O2—C15	1.4190 (14)	C8—H8	0.96
N1—C1	1.4555 (10)	C9—C10	1.3821 (13)
N1—C3	1.4696 (13)	C10—H10	0.96
N1—C4	1.4736 (11)	C11—C12	1.5358 (14)
C1—H1a	0.96	C11—C13	1.5353 (14)
C1—H1b	0.96	C11—C14	1.5306 (14)
C2—C3	1.5211 (12)	C12—H12a	0.96
C2—C3^i^	1.5211 (12)	C12—H12b	0.96
C2—H2a	0.96	C12—H12c	0.96
C2—H2b	0.96	C13—H13a	0.96
C3—H3a	0.96	C13—H13b	0.96
C3—H3b	0.96	C13—H13c	0.96
C4—C5	1.5100 (12)	C14—H14a	0.96
C4—H4a	0.96	C14—H14b	0.96
C4—H4b	0.96	C14—H14c	0.96
C5—C6	1.3993 (13)	C15—H15a	0.96
C5—C10	1.3966 (12)	C15—H15b	0.96
C6—C7	1.4103 (13)	C15—H15c	0.96
			
C6—O1—H1	104.8 (10)	C7—C8—H8	118.9115
C9—O2—C15	117.23 (8)	C9—C8—H8	118.9125
C1—N1—C3	110.33 (7)	O2—C9—C8	115.22 (8)
C1—N1—C4	110.60 (8)	O2—C9—C10	124.76 (9)
C3—N1—C4	111.94 (7)	C8—C9—C10	120.02 (8)
N1—C1—N1^i^	109.94 (10)	C5—C10—C9	119.37 (9)
N1—C1—H1a	109.4711	C5—C10—H10	120.3163
N1—C1—H1b	109.4713	C9—C10—H10	120.3168
N1^i^—C1—H1a	109.4711	C7—C11—C12	110.78 (8)
N1^i^—C1—H1b	109.4713	C7—C11—C13	109.09 (8)
H1a—C1—H1b	108.995	C7—C11—C14	112.16 (8)
C3—C2—C3^i^	110.20 (11)	C12—C11—C13	109.63 (8)
C3—C2—H2a	109.4711	C12—C11—C14	107.38 (8)
C3—C2—H2b	109.4714	C13—C11—C14	107.72 (8)
C3^i^—C2—H2a	109.4711	C11—C12—H12a	109.4717
C3^i^—C2—H2b	109.4714	C11—C12—H12b	109.472
H2a—C2—H2b	108.7329	C11—C12—H12c	109.4713
N1—C3—C2	109.43 (9)	H12a—C12—H12b	109.4696
N1—C3—H3a	109.4709	H12a—C12—H12c	109.4713
N1—C3—H3b	109.4712	H12b—C12—H12c	109.4714
C2—C3—H3a	109.4713	C11—C13—H13a	109.4718
C2—C3—H3b	109.4714	C11—C13—H13b	109.4714
H3a—C3—H3b	109.5159	C11—C13—H13c	109.4712
N1—C4—C5	112.83 (8)	H13a—C13—H13b	109.4709
N1—C4—H4a	109.4712	H13a—C13—H13c	109.4705
N1—C4—H4b	109.4728	H13b—C13—H13c	109.4715
C5—C4—H4a	109.4707	C11—C14—H14a	109.4717
C5—C4—H4b	109.4701	C11—C14—H14b	109.4711
H4a—C4—H4b	105.8863	C11—C14—H14c	109.4716
C4—C5—C6	120.76 (8)	H14a—C14—H14b	109.4711
C4—C5—C10	118.83 (8)	H14a—C14—H14c	109.4707
C6—C5—C10	120.29 (8)	H14b—C14—H14c	109.4712
O1—C6—C5	119.44 (8)	O2—C15—H15a	109.4715
O1—C6—C7	119.68 (8)	O2—C15—H15b	109.4712
C5—C6—C7	120.88 (8)	O2—C15—H15c	109.4719
C6—C7—C8	117.26 (8)	H15a—C15—H15b	109.4706
C6—C7—C11	121.53 (8)	H15a—C15—H15c	109.4704
C8—C7—C11	121.18 (8)	H15b—C15—H15c	109.4716
C7—C8—C9	122.18 (8)		
			
C15—O2—C9—C8	175.19 (9)	O1—C6—C7—C11	2.32 (13)
C15—O2—C9—C10	−4.54 (15)	C5—C6—C7—C8	0.56 (14)
C3—N1—C1—N1^i^	−62.71 (10)	C5—C6—C7—C11	−177.47 (9)
C4—N1—C1—N1^i^	172.88 (8)	C6—C7—C8—C9	−0.03 (15)
C1—N1—C3—C2	58.60 (11)	C11—C7—C8—C9	178.01 (9)
C4—N1—C3—C2	−177.78 (8)	C6—C7—C11—C12	−61.16 (11)
C1—N1—C4—C5	−165.79 (8)	C6—C7—C11—C13	59.62 (11)
C3—N1—C4—C5	70.73 (10)	C6—C7—C11—C14	178.86 (9)
C3^i^—C2—C3—N1	−54.82 (13)	C8—C7—C11—C12	120.88 (10)
N1—C4—C5—C6	43.06 (12)	C8—C7—C11—C13	−118.34 (10)
N1—C4—C5—C10	−140.83 (9)	C8—C7—C11—C14	0.91 (13)
C4—C5—C6—O1	−4.30 (14)	C7—C8—C9—O2	179.76 (9)
C4—C5—C6—C7	175.48 (9)	C7—C8—C9—C10	−0.50 (15)
C10—C5—C6—O1	179.64 (9)	O2—C9—C10—C5	−179.80 (10)
C10—C5—C6—C7	−0.58 (14)	C8—C9—C10—C5	0.49 (15)
C4—C5—C10—C9	−176.10 (9)	H1—O1—C6—C5	−16.4 (9)
C6—C5—C10—C9	0.03 (15)	H1—O1—C6—C7	163.8 (9)
O1—C6—C7—C8	−179.65 (8)		

Symmetry codes: (i) *x*, −*y*+1/2, *z*.

### Hydrogen-bond geometry (Å, °)

**Table:**

*D*—H···*A*	*D*—H	H···*A*	*D*···*A*	*D*—H···*A*
O1—H1···N1	0.890 (15)	1.843 (15)	2.6735 (10)	154.6 (14)
C12—H12a···O1	0.96	2.36	3.0103 (12)	124.92
C13—H13a···O1	0.96	2.38	2.9943 (12)	121.18
